# Curcumin in colorectal cancer: mechanistic insights, pharmacological limitations, and translational perspectives

**DOI:** 10.3389/fphar.2025.1667731

**Published:** 2025-09-29

**Authors:** Si-Qi Li, Xiao-Ren Zhu, Bai-Chun Qin, Min-Bin Chen

**Affiliations:** ^1^ Department of Radiotherapy and Oncology, Affiliated Kunshan Hospital of Jiangsu University, Kunshan, China; ^2^ Department of Development and Regeneration, Stem Cell Institute, Katholieke Universiteit (KU) Leuven, Leuven, Belgium; ^3^ Department of Gastrointestinal Surgery, Affiliated Kunshan Hospital of Jiangsu University, Kunshan, China

**Keywords:** curcumin, colorectal cancer, gut microbiota, immune response, signaling pathways, chemoresistance, pharmacological limitations, translational research

## Abstract

Curcumin, a natural polyphenolic compound from *Curcuma longa*, has been extensively investigated for its potential role in colorectal cancer (CRC) prevention and therapy. Preclinical studies suggest that curcumin can modulate gut microbiota composition, influence immune cell subsets such as M1/M2 macrophages, Treg/Th17 cells, and CD8^+^ T cells, and interfere with oncogenic signaling cascades including NF-κB, PI3K/Akt, and Wnt/β-catenin. These findings collectively highlight curcumin as a biologically active compound with broad mechanistic relevance. However, most evidence derives from *in vitro* assays at supra-physiological concentrations or high-dose animal models, raising concerns about pharmacological validity and clinical translatability. Curcumin is also recognized as a pan-assay interfering compound (PAINS), which may account for part of its pleiotropic activity and complicates interpretation of preclinical findings. Clinical trials to date have largely confirmed safety and biomarker modulation but have not demonstrated clear improvements in progression-free or overall survival. In this review, we critically appraise the available preclinical and clinical evidence on curcumin in CRC, highlighting both its mechanistic promise and the substantial limitations that constrain its therapeutic relevance, while outlining priorities for future research.

## 1 Introduction

Colorectal cancer (CRC) is one of the most prevalent malignant tumors of the digestive system and continues to pose a major public health burden worldwide (Siegel et al., 2023). Despite advances in screening, surgery, radiotherapy, and chemotherapy that have improved overall survival, patients with advanced-stage disease still face poor outcomes, characterized by recurrence, metastasis, and limited response to conventional treatments (Nussbaum et al., 2022; Doxtater and Tripathi, 2023). Tumor heterogeneity, therapy resistance, and immune escape remain significant barriers, highlighting the urgent need for innovative and well-tolerated strategies (Lv et al., 2023). CRC pathogenesis extends far beyond isolated genetic mutations or uncontrolled proliferation. Instead, it reflects a complex interplay between gut microbiota, immune responses, and key oncogenic signaling pathways. The gut microbiota, the largest microbial ecosystem in humans, shapes host immunity and mucosal barrier function through metabolites such as short-chain fatty acids (SCFAs) and secondary bile acids (Dong et al., 2023; Hays et al., 2024). Dysbiosis not only promotes chronic inflammation but also reprograms immune surveillance, thereby fueling tumor progression (Yang et al., 2023a). Meanwhile, aberrant activation of signaling cascades such as NF-κB, Wnt/β-catenin, and PI3K/Akt orchestrates malignant transformation, stemness maintenance, and metastatic dissemination, often in close interaction with microbiota–immune crosstalk.

In this context, polyphenolic compounds from natural products have drawn increasing attention due to their multi-targeted actions and favorable safety profiles. Curcumin, the principal bioactive constituent of *Curcuma longa*, has long been used in traditional medicine and is now extensively investigated as an anticancer agent (Kunnumakkara et al., 2008; Wilken et al., 2011; Li et al., 2020). Preclinical studies suggest that curcumin induces apoptosis, inhibits invasion, remodels the tumor microenvironment, and modulates both microbiota and immune populations. It has been reported to promote beneficial bacteria (e.g., *Lactobacillus* and Bifidobacterium) while suppressing pathogenic taxa such as *Clostridium difficile* and *Bacteroides fragilis*, thereby indirectly influencing immune tone and tumor progression (Lamichhane et al., 2024; Zhu and He, 2024).

However, enthusiasm must be tempered by recognition of important limitations. Much of the mechanistic evidence derives from *in vitro* studies employing supra-physiological concentrations, often orders of magnitude above achievable plasma levels in humans. Moreover, curcumin has been classified as a pan-assay interfering compound (PAINS), raising the possibility that some reported anticancer activities reflect non-specific or artifactual effects rather than true pharmacological actions (Nelson et al., 2017). Clinical trials to date confirm safety and modest biomarker modulation but provide limited evidence for survival benefit, largely due to small sample sizes, heterogeneous cohorts, and underpowered study designs. Against this background, the present review aims to provide a critical appraisal of curcumin’s potential in CRC, integrating evidence across the microbiota–immune–signaling axis. We emphasize both mechanistic promise and pharmacological uncertainties, evaluate preclinical and clinical evidence with attention to dose ranges, models, and methodological rigor, and outline future directions for improving bioavailability, refining study designs, and strengthening translational relevance.

## 2 Mechanism of CRC development and challenges in precision treatment

CRC is a heterogeneous malignancy that progresses through a multistep adenoma–carcinoma sequence driven by genetic mutations, epigenetic modifications, chronic inflammation, and microbial dysbiosis (Okugawa et al., 2015; Seligmann et al., 2022; Fakih et al., 2023; Storandt et al., 2023). High-throughput omics technologies have revealed CRC to be far more complex than a linear genetic model, highlighting its molecular heterogeneity and the interplay of signaling, immune regulation, and gut microbiota within the tumor microenvironment (TME). While this growing knowledge has improved molecular classification, its direct impact on treatment strategies remains constrained by variability in study design and translational gaps.

### 2.1 Molecular mechanisms and precision therapy challenges

**FIGURE 1:**
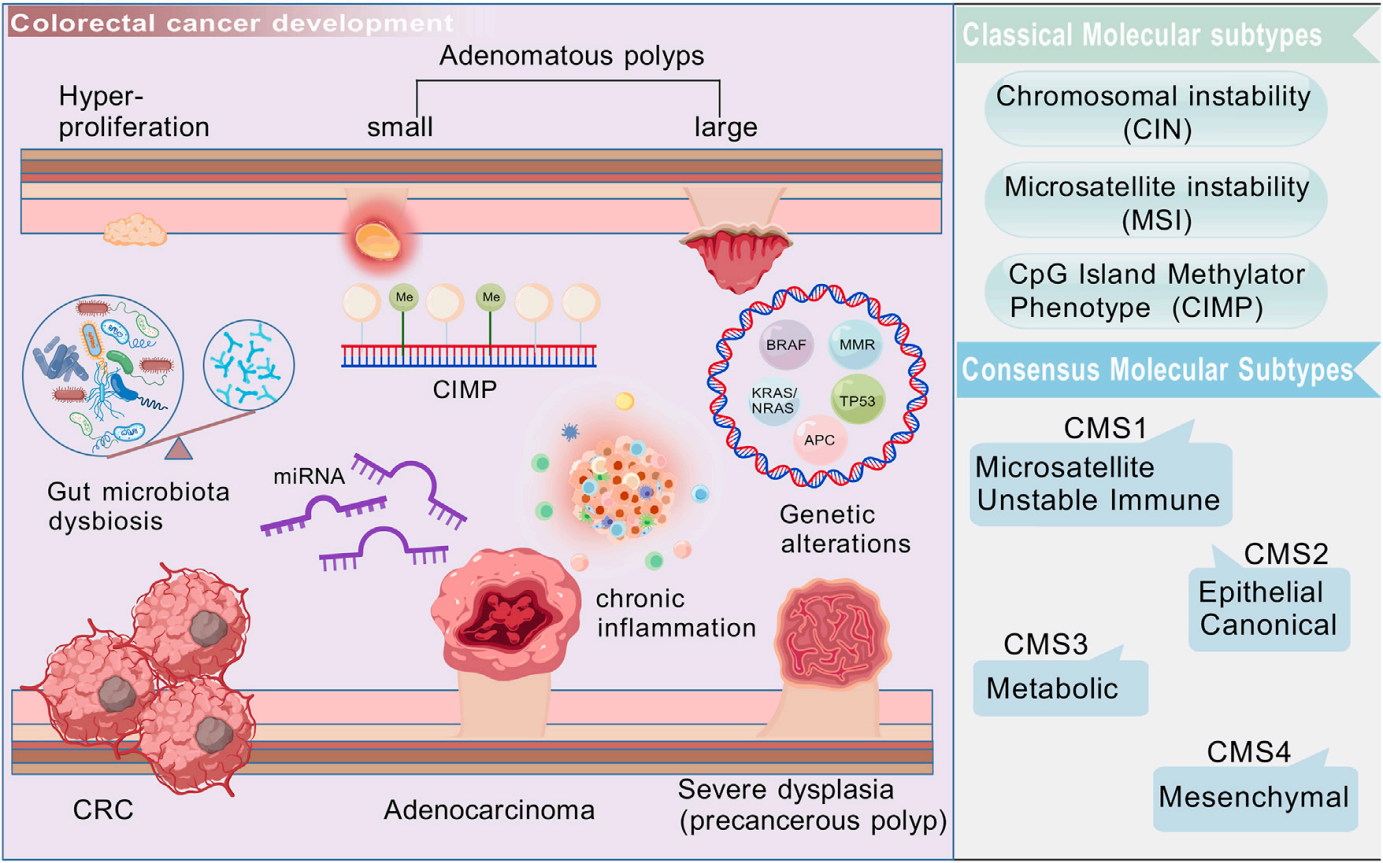
Colorectal cancer is a heterogeneous malignant tumor, originating from epithelial cells in the colon or rectum. Its development is a multistep process that involves the transformation of adenomatous polyps into invasive cancer, accompanied by complex pathological processes, such as gene mutations, epigenetic changes, inflammation, microenvironment shaping, and dysbiosis of gut microbiota. Created with BioGDP.com.

CRC development is classically explained by chromosomal instability (CIN), microsatellite instability (MSI), and CpG island methylator phenotype (CIMP) (Figure 1). CIN accounts for approximately 85% of sporadic CRC cases, involving APC and TP53 loss as well as KRAS or PIK3CA activation, which drive aberrant Wnt/β-catenin and PI3K/Akt signaling (Kudryavtseva et al., 2016; Hoevenaar et al., 2020; Al-Joufi et al., 2022). MSI, resulting from mismatch repair deficiency, occurs in 10%–15% of cases, and is associated with hypermutability and responsiveness to immune checkpoint blockade (The Cancer Genome Atlas Network, 2012; Dienstmann et al., 2017; Le et al., 2017). CIMP is characterized by widespread promoter methylation, frequently linked to BRAF mutations (Ichimura et al., 2015). The Cancer Genome Atlas (TCGA) initially provided a comprehensive molecular characterization of colorectal cancer (The Cancer Genome Atlas Network, 2012), and subsequent integrative analyses proposed four consensus molecular subtypes (CMS1–4) with distinct signaling, immune, and stromal profiles (The Cancer Genome Atlas Network, 2012; Guinney et al., 2015).

Although these classifications are valuable for research and biomarker discovery, their pharmacological relevance is still limited. Many studies report associations between CMS subtypes and treatment responses, yet most are retrospective and lack prospective stratification in clinical trials. Consequently, while molecular subtyping refines our understanding of CRC biology, its direct utility in precision therapy is still under active validation.

### 2.2 Gut microbiota in CRC development

Gut microbiota exerts profound effects on CRC through both protective and pathogenic species. Enrichment of *Fusobacterium* nucleatum, *Bacteroides fragilis*, and colibactin-producing *Escherichia coli* promotes tumorigenesis via TLR signaling, bacterial toxins, and barrier disruption (Lopez et al., 2021). Conversely, metabolites such as short-chain fatty acids (SCFAs) enhance mucosal immunity, modulate Treg activity, and inhibit HDACs, conferring anti-inflammatory and anti-tumor effects (Fellows et al., 2018; Mann et al., 2024). Secondary bile acids such as deoxycholic acid, however, can promote tumorigenesis by inducing oxidative stress and activating Wnt signaling (Ocvirk and O’Keefe, 2021).

Yet, most of these findings are derived from murine models or correlative human studies, where confounding factors such as diet and antibiotic use complicate interpretation. Moreover, metabolite concentrations reported to inhibit cancer cell growth *in vitro* often exceed physiological levels achievable *in vivo*. Therefore, while the microbiota is increasingly recognized as a driver and modifier of CRC, the causal mechanisms and pharmacological relevance in humans require more rigorous validation.

### 2.3 Tumor microenvironment and immune imbalance

The CRC tumor microenvironment (TME) integrates cancer cells with stromal fibroblasts, endothelial cells, immune infiltrates, and soluble mediators. Early immune surveillance can suppress tumor initiation, but tumors progressively achieve immune escape by recruiting Tregs and MDSCs, polarizing macrophages toward the M2 phenotype, and inducing T-cell exhaustion through PD-L1 and other checkpoints (Lei et al., 2020; Jancewicz et al., 2021). Effector populations such as CD8^+^ T cells and NK cells become functionally suppressed, while the balance between Th1/Th17 cells and Tregs strongly influences prognosis (Liu et al., 2021a; Calvo-Barreiro et al., 2023).

Despite extensive mechanistic insights, much of the evidence originates from murine models with limited comparability to human TME complexity. Importantly, the minimal effective concentrations of immunomodulatory factors or interventions are seldom reported, making pharmacological translation uncertain. Thus, while TME reshaping represents an attractive target, more rigorous studies with standardized immune readouts and clinically relevant dosing are needed.

### 2.4 Bottlenecks in precision medicine

Precision oncology has introduced immune checkpoint inhibitors (ICIs) and targeted therapies that yield remarkable benefits in defined CRC subgroups, such as MSI-high tumors (Le et al., 2017). However, heterogeneity remains a major barrier: CMS4 tumors, characterized by stromal activation and immune exclusion, show poor response to ICIs (Becht et al., 2016; Dienstmann et al., 2017), while accumulating evidence indicates that variations in gut microbiota strongly influence immunotherapy outcomes (Gopalakrishnan et al., 2018; Matson et al., 2018). Predictive biomarkers such as CD8^+^ T-cell infiltration, MSI status, or microbial composition are promising, but lack standardized validation across trials. Moreover, clinical trial evidence is often weakened by small sample sizes, heterogeneous cohorts, short follow-up, and inconsistent formulations of investigational agents such as curcumin. Microbiota profiling methodologies also vary widely, limiting reproducibility. Finally, immune escape mechanisms are highly redundant, suggesting that multi-target approaches will be required. These limitations highlight the gap between conceptual advances and clinical translation in CRC precision medicine.

## 3 Basic characteristics of curcumin and its anti-CRC mechanism: from signaling pathways to microecological remodeling

Curcumin has been extensively investigated for its anti-inflammatory, antioxidant, anticancer, immunomodulatory, and neuroprotective properties (Boroumand et al., 2018; Memarzia et al., 2021). In the CRC, curcumin’s therapeutic potential is not limited to direct inhibition of tumor proliferation or induction of apoptosis; preclinical studies show it also remodels the tumor microenvironment, modulates immune responses, and restores gut microbiota composition and metabolite profiles (Deng et al., 2024; Shakhpazyan et al., 2024; Zhou et al., 2025). This section reviews the structural basis and pharmacological activities of curcumin, focusing on its capacity to modulate oncogenic signaling pathways, immune responses, and intestinal microecology.

### 3.1 Structural characteristics and pharmacological activities of curcumin

Curcumin (C_21_H_20_O_6_) consists of two aromatic rings linked by an α,β-unsaturated β-diketone moiety (Priyadarsini, 2014). This conjugated structure underlies its strong resonance and free radical-scavenging ability, conferring antioxidant and anti-inflammatory effects. However, curcumin’s pharmacological utility is constrained by poor water solubility, low oral bioavailability, and rapid metabolic clearance. To address these challenges, advanced formulations—such as nano-emulsions, liposomes (Ternullo et al., 2019), solid lipid nanoparticles (Mulik et al., 2012; Yeo et al., 2022), cyclodextrin inclusion complexes, and phospholipid complexes (Maleki Dizaj et al., 2022)—have been developed. These systems significantly improve stability, absorption, and tissue targeting, though their comparative efficacy across clinical settings remains under evaluation. Importantly, pharmacological conclusions about curcumin’s activity must always be interpreted in light of formulation-dependent differences in bioavailability.

### 3.2 Regulatory roles in anti-tumor signaling pathways

**FIGURE 2:**
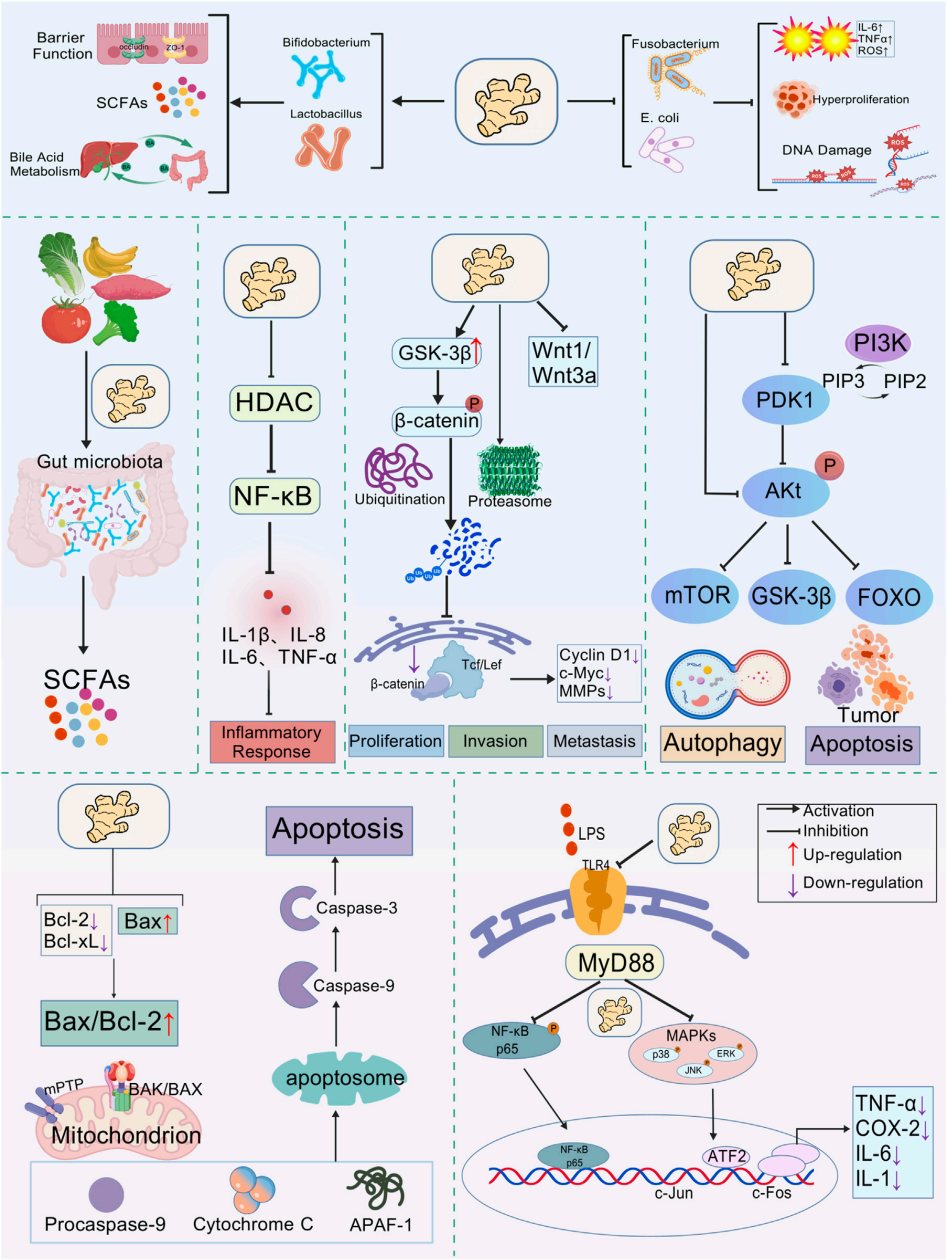
Multi-target mechanism framework of curcumin’s antitumor effects. Curcumin promotes the proliferation of beneficial gut bacteria (such as *Lactobacilli* and *Bifidobacteria*) while inhibiting pathogenic microorganisms (such as *Escherichia coli* and *Fusobacterium*). This effect is achieved by regulating the Bax/Bcl-2 balance and inhibiting carcinogenic signaling pathways (NF-κB, PI3K/AKT, Wnt/β-catenin), thus inducing tumor cell apoptosis and autophagy. Created with BioGDP.com.

Aberrant signaling is a hallmark of CRC, and curcumin is capable of intervening in several key pathways (Figure 2). The PI3K/Akt/mTOR axis, frequently activated in CRC, drives proliferation, survival, and therapy resistance (Stefani et al., 2021). Curcumin suppresses this pathway by inhibiting PI3K p110α and Akt phosphorylation, thereby downregulating mTOR activity and sensitizing resistant cells to chemotherapy (Chen et al., 2023; Deswal et al., 2024). While this suggests strong chemosensitizing potential, most evidence derives from *in vitro* studies at micromolar concentrations, which raises questions about conversion correlations. The Wnt/β-catenin pathway, central to stemness and metastasis in CRC, is another target. Curcumin accelerates β-catenin degradation and reduces downstream c-Myc and cyclin D1 expression, impairing clonogenicity and tumor-initiating capacity (Le and Kim, 2019; Liao et al., 2023). This mechanistic rationale is compelling, yet *in vivo* evidence remains limited, and dose-response relationships are not consistently defined.

The TLR4/MyD88/NF-κB pathway, which links microbial dysbiosis to chronic inflammation and carcinogenesis, is also suppressed by curcumin. It blocks TLR4 recognition of pathogenic ligands, prevents NF-κB activation, and reduces pro-inflammatory mediators such as IL-6, TNF-α, and COX-2 (Boozari et al., 2019; Guo et al., 2021). Although these findings support the concept of an “anti-inflammatory shield,” they often rely on models with artificial stimulation or supraphysiological curcumin exposure. Therefore, while curcumin’s multi-targeted signaling modulation is well established, the pharmacological strength of evidence varies depending on the model system and achievable concentrations.

### 3.3 Tumor immune regulatory effects

Curcumin exerts multi-faceted immunomodulatory effects. It enhances CD8^+^ T-cell proliferation and cytotoxic activity, increasing perforin and granzyme B secretion (Liu et al., 2021b). Furthermore, it rebalances CD4^+^ T-cell subsets by reducing Tregs and promoting Th1/Th17 responses (Zou et al., 2018; Shafabakhsh et al., 2019; Fu et al., 2021). Particularly noteworthy is the ability of curcumin to convert Foxp3^+^ Tregs into Th1-like cells, thereby reinforcing anti-tumor immunity (Zou et al., 2018). Macrophage polarization is another critical target, CRC TMEs are enriched with pro-tumorigenic M2 macrophages, while curcumin suppresses M2 markers (IL-10, ARG1) and activates STAT6/MAO-A-related switches, shifting toward an M1 phenotype (Jiang et al., 2022).

Nevertheless, immune regulation by curcumin is context- and dose-dependent. *In vitro*, curcumin often suppresses dendritic cell (DC) maturation, reducing co-stimulatory molecule expression and T-cell priming capacity (Shirley et al., 2008). In contrast, *in vivo* studies using high-bioavailability formulations demonstrate enhanced DC antigen presentation via STAT3 inhibition (Hayakawa et al., 2020). These conflicting results underscore the importance of formulation, concentration, and biological context, and highlight the need for dose-optimized strategies in clinical applications.

### 3.4 Gut microbiota remodeling and immune-metabolic regulation

Curcumin also exerts its effects indirectly by remodeling gut microbiota. It increases beneficial genera such as Bifidobacterium and *Lactobacillus* while reducing pro-inflammatory taxa such as *Fusobacterium*, Prevotella, and Enterobacteriaceae (Pluta et al., 2020; Zhu and He, 2024). These changes shift microbial metabolism toward greater short-chain fatty acid (SCFA) production, particularly butyrate, which supports epithelial energy metabolism, reinforces barrier function, and epigenetically regulates T-cell differentiation (Van Deuren et al., 2022). Curcumin also strengthens barrier integrity by upregulating tight junction proteins (ZO-1, occludin, claudin-1), thereby limiting lipopolysaccharide (LPS) translocation and systemic inflammation (Burge et al., 2019; Cao et al., 2020). This creates a feedback loop in which microbiota restoration, metabolic regulation, and immune modulation act synergistically to restrain tumorigenesis.

However, the majority of microbiota studies rely on rodent models, with results influenced by diet, housing, and antibiotic exposure. Human evidence is relatively sparse, and mechanistic links remain associative rather than causal. Moreover, the concentrations of curcumin required to shift microbial communities are not always aligned with pharmacologically achievable levels. Therefore, while gut microbiota remodeling is a promising dimension of curcumin’s activity, its clinical translation requires rigorous validation through standardized multi-omics studies and controlled interventions.

## 4 Progress in preclinical and translational research on curcumin

Despite extensive research into the molecular mechanisms underlying curcumin’s anti-CRC effects, its clinical translation ultimately depends on the robustness of preclinical evidence across multiple experimental levels. Chemically induced CRC models, such as the azoxymethane/dextran sulfate sodium (AOM/DSS) inflammation-associated system, have consistently demonstrated that oral or gavage administration of curcumin reduces tumor number and volume, alleviates mucosal ulceration, and decreases dysplastic gland formation (Deng et al., 2024). Mechanistic analyses highlight suppression of the TLR4/MyD88–NF-κB pathway and downregulation of inflammatory mediators, including IL-6, TNF-α, and COX-2 (Buhrmann et al., 2019; Liu et al., 2020; Iglesias et al., 2022; Yang et al., 2023b). These results provide strong biological plausibility for curcumin as an inflammation-modulating agent. However, it must be emphasized that the doses applied in rodents (often >100 mg/kg/day) are much higher than typical human exposures, and the curcumin formulations used are heterogeneous, limiting extrapolation to human pharmacology (Anand et al., 2007; Sharma et al., 2007).

In xenograft models using human CRC cells, curcumin has been shown to suppress tumor growth and enhance sensitivity to 5-fluorouracil (5-FU), oxaliplatin, and irinotecan (Howells et al., 2019; Zhao et al., 2020; Zheng et al., 2021). Mechanistically, this effect is associated with inhibition of PI3K/Akt signaling (Astinfeshan et al., 2019; Jin et al., 2021; Liu et al., 2021b), suppression of cancer stemness markers (SOX2, OCT4, CD44, CD133, LGR5), and attenuation of IL-6/STAT3-and NF-κB-driven survival signaling (Prud’homme, 2012; Hu et al., 2019; Brockmueller et al., 2023; Kubatka et al., 2024). Furthermore, curcumin downregulates anti-apoptotic proteins and modulates ATP-binding cassette (ABC) transporters, such as P-glycoprotein (P-gp), thereby reducing drug efflux and increasing intracellular concentrations of chemotherapeutics (Fathy Abd-Ellatef et al., 2020). These findings suggest a strong chemosensitizing role for curcumin. Yet, most xenograft studies involve immunodeficient mice that lack a functional adaptive immune system (Sharma et al., 2005; Olive and Tuveson, 2006), preventing evaluation of immune-dependent mechanisms that are central to human CRC biology. Moreover, the curcumin concentrations applied *in vitro* (often 10–50 μM) far exceed clinically achievable plasma levels (<1 μM), raising questions about translational pharmacological relevance (Anand et al., 2007; Sharma et al., 2007; Wilken et al., 2011).

Patient-derived tumor organoids (PDOs) represent an important step toward bridging preclinical and clinical research. Studies have shown that even relatively low micromolar concentrations of curcumin can inhibit organoid proliferation, suppress ERK phosphorylation, and reduce expression of stemness markers such as CD44, CD133, and LGR5 (Elbadawy et al., 2021). Compared with cell-line xenografts, organoids preserve inter-patient heterogeneity and more closely mimic clinical tumor responses, making them attractive platforms for personalized drug testing and screening of combination regimens. However, PDO studies remain scarce, with small sample sizes and variable culture conditions, limiting their generalizability. In addition, most work has focused on acute responses (≤72 h) rather than long-term resistance dynamics, which may underestimate the challenges of clinical translation (Elbadawy et al., 2021; Furbo et al., 2022).

Collectively, preclinical evidence consistently indicates that curcumin exerts anti-CRC effects through multi-targeted signaling suppression, immune modulation, and chemosensitization. Nevertheless, the majority of findings are constrained by supraphysiological dosing, variability in curcumin formulations, limited immune-competent models, and small-scale PDO studies. Thus, while curcumin emerges as a promising adjunct or synergistic therapy, rigorous pharmacological assessment—encompassing standardized dosing protocols, bioavailability optimization, immune-competent animal studies, and biomarker-guided patient stratification—is required before reliable clinical translation can be achieved.

## 5 Limitations and pharmacological appraisal of curcumin in CRC

Although curcumin has been widely studied for its potential in CRC, a rigorous pharmacological appraisal reveals important limitations that temper enthusiasm for its therapeutic translation (Table 1). Without such critical analysis, there is a risk of overestimating curcumin’s value based on preclinical findings alone. A first concern relates to curcumin’s chemical nature as a PAINS (Baker, 2017; Nelson et al., 2017; Padmanaban and Nagaraj, 2017). Such compounds are notorious for producing false-positive results in diverse bioassays due to intrinsic properties such as redox activity, fluorescence interference, nonspecific protein binding, or covalent modification of nucleophilic residues. These features complicate the interpretation of experiments that report modulation of key signaling pathways, including NF-κB, PI3K/Akt, Wnt/β-catenin, and STAT3. Many of the supposed “multi-targeted” effects may reflect assay artifacts rather than genuine pharmacological specificity. Unless confirmed through orthogonal assays or validated *in vivo*, such mechanistic claims should be regarded with caution.

**TABLE 1:** Critical pharmacological appraisal of curcumin studies in CRC.

Study type	Dose/exposure (reported)	HED (mg/kg/day)	Key limitations	Evidence strength	Reference
*In vitro* — CRC cell lines (mechanistic, chemosensitization)	Curcumin 5–20 μM; many reported effects at ≥10 μM; exposures typically 24–72 h	N/A	Concentrations exceed clinically achievable plasma (<∼1–2 μM after oral dosing); MAC not reported; IC_50_ partially reported; PAINS/assay interference possible	Weak — mechanistic only; poor direct translational relevance	Shakibaei et al. (2014), Toden et al. (2015)
*Ex vivo* — patient-derived explants (CRC liver metastases)	Curcumin ≈5 μM ± 5-FU (5 μM) or oxaliplatin (2 μM); 24–72 h	N/A	Short-term culture (*ex vivo*); no systemic metabolism (*ex vivo*); limited explant numbers; heterogeneous responses; Phase I trial small sample size (n = 12), no efficacy comparison with FOLFOX alone	Moderate — mechanistic chemosensitization evidence (*ex vivo*) + clinical safety/tolerability data, but lacks systemic PK confirmation and large-scale efficacy validation	James et al. (2015)
PDOs — patient-derived organoids	*In vitro* (PDOs): Amorphous curcumin (AC) 0.6–20 μg/mL (not μM); exposures 24–72 h; combined with oxaliplatin (0.1–100 μg/mL), 5-FU (0.1–100 μg/mL) or irinotecan (0.1–100 μg/mL); *In vivo* (xenografts): AC 20 mg/mouse/day (oral gavage), administered for 21 days	*In vivo* AC: ∼56 mg/kg/day; *In vitro* (PDOs): N/A	Limited PDO panels; formulation-dependent effects; short exposure windows (*in vitro*); *in vivo* anti-tumor effect diminished after day 14 (possible due to short AC half-life); no clinical data	Moderate — more physiologically relevant than cell lines, but translational gap remains (no clinical validation)	Elbadawy et al. (2021)
Animal models (xenograft)	Curcumin (C3 complex): 1 g/kg/day (oral, once daily); γ-radiation: 4 Gy, twice weekly (given 1 h after curcumin for combination group); treatment duration up to 30 days	HED ≈83.3 mg/kg/day	Animal doses exceed clinically feasible oral regimens; many use enhanced formulations; xenografts in immunodeficient hosts; limited tumor/tissue PK	Moderate — reproducible tumor suppression in animals; limited human translatability without PK/PD	Kunnumakkara et al. (2008)
Phase I PK/PD clinical studies	Oral curcumin 0.45–3.6 g/day (C3 extract)	≈7.5–60 mg/kg/day	Very small cohorts; low systemic free curcumin (mostly conjugates); surrogate endpoints (GST activity, M1G adducts, PGE_2_)	Moderate — confirms safety, limited PD activity	Sharma et al. (2004), Garcea et al. (2005)
Phase IIa biomarker/chemoprevention trials (ACF, FAP pilot)	Oral 2 or 4 g/day (ACF trial); FAP pilot: curcumin 480 mg + quercetin 20 mg TID (∼1.44 g/day curcumin)	ACF trial:≈ 33–67 mg/kg/day; FAP pilot: ≈24 mg/kg/day	Very small sample size; surrogate endpoints (ACF counts, polyp burden); short follow-up	Moderate — biomarker modulation observed but clinical benefit unproven	Cruz–Correa et al. (2006), Carroll et al. (2011)
Randomized clinical trials	Oral curcumin (C3 Complex) 2 g/day; FOLFOX chemotherapy given every 2 weeks for ≤12 cycles; treatment duration until disease progression, toxicity, or withdrawal	≈33 mg/kg/day	Underpowered trials; heterogeneous formulations/regimens; variable endpoints; efficacy neutral/negative	Moderate — safety supported; OS improved in CUFOX but limited by small sample size and baseline imbalances; no consistent PFS benefit	Howells et al. (2019)

The limitations become even more evident when examining *in vitro* studies. Anti-proliferative or chemosensitizing effects of curcumin in CRC cell lines such as HCT116, HT-29, or SW480 are typically observed at concentrations between 5 and 50 μM, with minimal active concentrations rarely below 5 μM (Link et al., 2013; Li et al., 2021; Yang et al., 2022). Yet, pharmacokinetic studies consistently show that even with oral administration of 8–12 g/day in humans, plasma concentrations seldom exceed 1–2 μM, and circulating curcumin largely exists in conjugated forms rather than as free active compound (Vareed et al., 2008; Urošević et al., 2022). This discrepancy underscores a key translational gap, many effects reported *in vitro* are unlikely to occur *in vivo* at physiologically achievable exposures. Furthermore, *in vitro* experiments often lack rigorous pharmacological controls, with positive and negative comparators inconsistently included and standardized pharmacodynamic parameters such as IC_50_ or therapeutic index rarely reported. The heterogeneity of exposure times—ranging from short-term assays to long-term clonogenic models—further complicates interpretation and limits extrapolation to clinical contexts.

Animal models provide supportive but similarly constrained evidence. Xenograft models using immunodeficient mice have demonstrated that curcumin can inhibit tumor growth or enhance the effects of chemotherapy, yet these studies often rely on doses such as 100 mg/kg (Shaikh et al., 2021), which correspond to several grams per day in human equivalent dosing, levels not feasible in clinical practice. In addition, many animal studies employ formulations with artificially enhanced bioavailability, including nanoparticles or curcumin–piperine combinations, which are not consistently available or standardized for human use. A further limitation is the lack of pharmacokinetic monitoring in tumor tissues, making it unclear whether observed effects are attributable to direct tumor exposure or to systemic anti-inflammatory activity (Dhillon et al., 2008; Ozawa-Umeta et al., 2020). The frequent reliance on immunodeficient hosts also omits critical contributions of immune and microbiota pathways, both of which are proposed to be central to curcumin’s mechanisms in CRC.

Clinical trials, though offering the most relevant data, remain small, heterogeneous, and often underpowered. Early pharmacokinetic and pharmacodynamic studies confirmed that curcumin is safe at doses up to 3.6 g/day and demonstrated reductions in biomarkers such as DNA adducts, but these were limited to fewer than 20 patients and focused on surrogate endpoints of uncertain predictive value (Sharma et al., 2004; Garcea et al., 2005). Phase IIa studies, including those assessing aberrant crypt foci (ACF) or familial adenomatous polyposis (FAP), suggested modest benefits but were limited by small sample sizes and endpoints not directly linked to long-term clinical outcomes (Carroll et al., 2011). More recent studies combining curcumin with chemotherapy in metastatic CRC have shown acceptable safety but no clear improvements in progression-free or overall survival, while randomized placebo-controlled trials in locally advanced rectal cancer failed to demonstrate clinical benefit and in some cases even suggested numerically worse complete response rates in curcumin-treated groups (Gunther et al., 2022). These results highlight a striking disconnect between preclinical promise and clinical reality.

Taken together, the current body of evidence illustrates that while curcumin is biologically active in CRC-related systems, its translation into clinically meaningful efficacy remains unproven. The PAINS nature of curcumin raises the possibility of false-positive mechanistic findings, *in vitro* studies rely on supraphysiological concentrations, animal models use doses or formulations not applicable to patients, and clinical trials remain exploratory and inconclusive. This evidence landscape underscores the need for a more rigorous and standardized research framework. Future work should emphasize dose–response characterization, tissue-level pharmacokinetic–pharmacodynamic correlation, the use of clinically relevant and immunocompetent models, and adequately powered randomized trials with standardized formulations and hard endpoints such as progression-free survival and overall survival. Equally important is the incorporation of biomarker-driven patient stratification, allowing identification of subgroups most likely to benefit from curcumin-based interventions. In summary, curcumin research in CRC offers both opportunities and cautionary lessons. It demonstrates the potential of natural products to modulate complex biological systems, but also illustrates the risks of overinterpreting descriptive or artifact-prone data. Only by adopting pharmacologically rigorous and clinically robust approaches can the field determine with confidence whether curcumin holds genuine therapeutic relevance for colorectal cancer.

## 6 Future directions

Although curcumin has long been celebrated as a natural product with broad pharmacological potential, its clinical translation in CRC continues to face major obstacles. The accumulated literature demonstrates that curcumin can influence the microbiota–immune–signaling axis and reshape the tumor microenvironment, yet the strength of these findings remains uncertain. A critical challenge arises from the recognition that curcumin belongs to the class of PAINS, which are notorious for producing assay artifacts and nonspecific signals in biochemical and cellular experiments. This raises the risk that many of the reported “anticancer” effects may reflect *in vitro* artifacts rather than pharmacologically meaningful mechanisms. Future research must therefore adopt a more rigorous and hypothesis-driven approach to establish the true scope and relevance of curcumin’s biological activity.

One of the most pressing needs is to move beyond descriptive evidence toward rigorous pharmacological standards. Current reports often claim that curcumin inhibits NF-κB activation, regulates CD8^+^ T cells, or alters gut microbiota composition, but such conclusions are frequently unsupported by systematic dose–response analyses or pharmacokinetic validation. The use of concentrations far above clinically achievable levels undermines translational credibility. It is essential that future studies define minimal effective concentrations, establish concentration–effect curves, and incorporate appropriate controls to exclude nonspecific PAINS-related effects (Nelson et al., 2017). Moreover, detailed reporting of formulation, purity, and delivery systems must become standard to enable reproducibility and cross-study comparison. Without this level of rigor, mechanistic claims risk remaining anecdotal rather than clinically actionable. Equally important is the need to bridge the persistent gap between *in vitro* assays and *in vivo* relevance. Curcumin’s proposed mechanisms of action, ranging from immune modulation to microbiota reshaping, cannot be meaningfully assessed in conventional tumor cell lines alone. More advanced models are required, including patient-derived organoids co-cultured with immune components, immune-competent mouse models, and germ-free or microbiota-humanized systems that capture the host–microbiota–immune interplay. These platforms will help clarify whether curcumin’s observed effects reflect direct cytotoxicity, immunomodulation, microbial reshaping, or artifacts of simplified assay systems. Without this integration, the field risks overinterpreting results from reductionist models. Another critical issue lies in curcumin’s unfavorable pharmacokinetic profile. Poor solubility, extensive metabolism, and rapid clearance limit systemic exposure, raising the paradox of how curcumin exerts strong *in vitro* activity at concentrations never attained in human plasma. Although formulation strategies such as nanoparticles, liposomes, and phytosomal complexes have improved bioavailability, systematic pharmacokinetic/pharmacodynamic (PK/PD) correlations remain rare. Future research should consistently report plasma and tissue concentrations alongside pharmacodynamic readouts, calculate human equivalent doses when extrapolating from animal models, and explore localized delivery systems that exploit curcumin’s potential activity in the gut mucosa without requiring high systemic levels. Only through this type of pharmacological discipline can the field resolve the disconnect between laboratory efficacy and clinical plausibility.

Clinical evidence to date, while encouraging in isolated trials, remains fragmented and underpowered. Small sample sizes, heterogeneous formulations, and inconsistent endpoints have limited the interpretability of curcumin trials in CRC. Future studies must be designed as adequately powered, biomarker-driven randomized controlled trials, ideally stratifying patients by molecular subtype, immune contexture, or microbiota composition. Integration of multi-omics biomarkers into trial design could transform exploratory observations into precision-guided interventions, identifying patient subgroups most likely to benefit. Equally important is the use of standardized clinical endpoints such as progression-free survival, overall survival, and validated biomarker changes, rather than reliance on surrogate markers alone.

Avoiding the PAINS trap also requires cultural change within the field. Researchers must implement PAINS-aware assay design, including aggregation counterscreens and orthogonal validation methods. Furthermore, rather than continuing to portray curcumin as a universal anticancer agent, future research should focus on contexts where its effects are most reproducible and mechanistically plausible, for example, in inflammation-associated CRC where microbiota modulation and mucosal barrier protection may be more relevant than direct tumor cytotoxicity.

**FIGURE 3:**
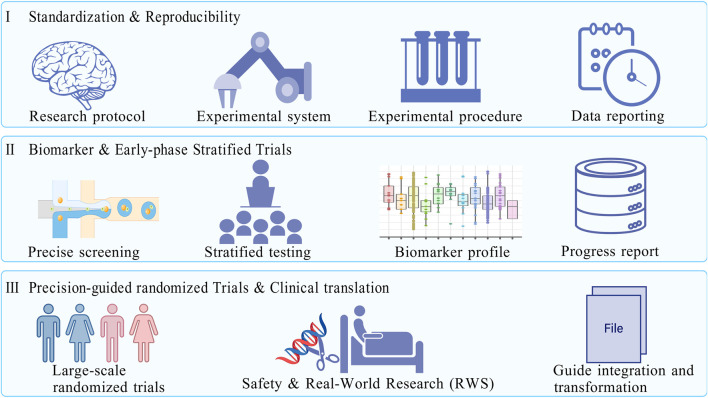
A phased roadmap for curcumin in colorectal cancer research. Based on the current status of curcumin research (safety verified, bioavailability and mechanism need to be deepened, and clinical evidence needs to be strengthened), the roadmap is divided into three research stages to promote the transformation of curcumin from basic research to clinical practice. Phase I: Focus on the standardization of experimental systems and cross-laboratory reproducibility verification; Phase II.: Explore predictive biomarkers related to curcumin efficacy, develop a “patient stratification strategy”, and carry out early clinical stratification trials with small samples and precise enrollment; Phase III: Conduct a large-scale precision guidance randomized trial (Phase III) to verify the survival benefits of curcumin combined with chemotherapy, and promote its inclusion in clinical guidelines in combination with real-world research to ensure research translation. Created with BioGDP.com.

Taken together, these considerations point to a research roadmap that prioritizes standardization and reproducibility in the near term, biomarker discovery and early-phase stratified trials in the medium term, and large-scale precision-guided randomized trials in the long term (Figure 3). Such a staged approach will allow the field to move from descriptive enthusiasm toward rigorous evidence-based evaluation. The ultimate goal is not merely to confirm whether curcumin has anticancer activity, but to define under what conditions, in which patient subgroups, and through which validated mechanisms such activity can be reliably observed. In conclusion, curcumin represents both a cautionary tale and a continuing opportunity in natural product research. Its wide-ranging mechanistic effects have attracted enormous scientific attention, yet the translational significance of these findings remains uncertain without stronger pharmacological foundations. By adopting rigorous assay standards, bridging the in vitro–in vivo gap, addressing pharmacokinetic limitations, and embedding biomarker-guided design into clinical research, the field can move beyond descriptive speculation and toward true precision medicine. Only under such conditions can curcumin be repositioned from a widely cited but weakly validated compound into a scientifically credible candidate for colorectal cancer prevention or therapy.
